# Usability of the Japanese Late‐Stage Elderly Questionnaire for screening major depression

**DOI:** 10.1111/psyg.13146

**Published:** 2024-06-05

**Authors:** So Sato, Yusuke Sasabuchi, Akira Okada, Hideo Yasunaga

**Affiliations:** ^1^ Department of Clinical Epidemiology and Health Economics, Graduate School of Medicine The University of Tokyo Tokyo Japan; ^2^ Department of Real‐World Evidence, Graduate School of Medicine The University of Tokyo Tokyo Japan; ^3^ Department of Prevention of Diabetes and Lifestyle‐Related Diseases, Graduate School of Medicine The University of Tokyo Tokyo Japan

**Keywords:** depression, geriatric depression scale, health check‐ups, Late‐Stage Elderly Questionnaire, predictive abilities

## Abstract

**Background:**

Older adults with major depression are at risk of frailty and long‐term care needs. Consequently, screening for major depression is imperative to prevent such risks. In Japan, the Late‐Stage Elderly Questionnaire was developed to evaluate older adults' holistic health, including mental well‐being. It comprises one specific question to gauge life satisfaction, but the effectiveness of this question to screen for major depression remains unclear. Therefore, we aimed to assess the usability of this question to screen for major depression.

**Methods:**

This retrospective cohort study used a large, commercially available claims database in Japan. Participants were older adults aged ≥75 years who completed the Late‐Stage Elderly Questionnaire and were classified with and without new major depression within 1 year. We evaluated the questionnaire's ability to screen for major depression using C‐statistics, developing three models to assess the cut‐off value based on responses to the life satisfaction question (‘Satisfied’, ‘Somewhat satisfied’, ‘Somewhat unsatisfied’, or ‘Unsatisfied’), estimating the sensitivity and specificity of each model.

**Results:**

Among 11 117 older adults, 77 newly experienced major depression within 1 year. The C‐statistic for screening major depression was 0.587. The model setting the cut‐off between ‘Somewhat unsatisfied’ and ‘Unsatisfied’ the demonstrated lowest sensitivity and highest specificity, while the model setting the cut‐off between ‘Satisfied’ and ‘Somewhat satisfied’ demonstrated highest sensitivity and lowest specificity.

**Conclusions:**

Our results suggest that due to its poor screening ability and high rate of false negatives, the question assessing life satisfaction in the Late‐Stage Elderly Questionnaire may not be useful for screening major depression in older adults and may require modification.

## INTRODUCTION

Using an appropriate depression screening tool for older adults is crucial because those with major depression are associated with frailty, an increased risk of long‐term care needs, and a decline in activities of daily living compared to those without major depression.^1^, ^2^ Nevertheless, diagnosing depression in older adults is difficult,^3^ and routine depression screening has become a topic of debate.^4^, ^5^ The United Kingdom National Screening Committee does not recommend the introduction of a systematic population screening program for depression due to uncertainties, such as whether such screening would effectively mitigate the negative impact of depression.^4^ Conversely, The United States Preventive Services Task Force recommends screening for major depression to mitigate the risks associated with untreated depression,^5^ including potential suicide^6^ and economic strain.^7^

The Geriatric Depression Scale comprising 15 questions (GDS‐15) is a screening tool for geriatric depression, having performed well in screening for major depression among older adults in various countries and settings.^8^, ^9^, ^10^, ^11^ While several studies have reported the ability of each question in the GDS to predict major depression in older adults, the results were inconsistent.^12^, ^13^, ^14^

To enhance the well‐being of older adults, Japan introduced the Late‐Stage Elderly Questionnaire in 2020 as a part of health checkups for older adults aged ≥75 years.^15^ Originally designed to screen for frailty, this questionnaire has been shown to comprehensively assess the health of older adults across physical, mental, and social aspects.^16^ A previous study has also suggested its usefulness in screening for frailty.^17^ In the mental health status section of the questionnaire, one question that addresses life satisfaction is identical to Item 1 of the original GDS‐15^18^ although it presents four response options as opposed to two in the GDS‐15.

No study, until now, has examined the ability of this question to screen for major depression. Therefore, our study seeks to bridge this research gap by assessing the effectiveness of the question in the mental health status section of the Late‐Stage Elderly Questionnaire to screen for major depression in older adults.

## METHODS

### Data source

We used the DeSC database (DeSC Healthcare, Inc., Tokyo, Japan), a large Japanese commercial medical claims and health checkup data repository. Detailed information on this database has been previously described.^19^, ^20^ The DeSC database includes health insurance claims data from three health insurer categories: (i) National Health Insurance for the self‐employed, irregularly employed, and pensioners <75 years; (ii) society‐managed health insurance for large companies; and (iii) Advanced Elderly Medical Service System for individuals aged ≥75 years. It contains information on approximately 12 000 000 individuals, and the age distribution within the DeSC database closely reflects estimates of the Japanese population.^20^ The claims data comprise the following information: (i) an anonymized identifier; (ii) birth year and month, and sex; (iii) diagnoses coded in accordance with the International Classification of Diseases, 10th Revision (ICD‐10) codes; (iv) procedures recorded based on original Japanese codes; (v) pharmaceutical dispensations documented using the Anatomical Therapeutic Chemical (ATC) Classification System; and (vi) insured dates for enrollment and disenrollment. Accurate mortality information can be obtained for those individuals who are covered by the National Health Insurance and Advanced Elderly Medical Service System.

Additionally, the database encompasses health checkup data. The Late‐Stage Elderly Questionnaire is administered to individuals aged 75 years or older as part of their annual health checkups. This self‐administered questionnaire is divided into 10 sections: (i) health status, (ii) mental health status, (iii) dietary habits, (iv) oral function, (v) weight change, (vi) physical function and falls, (vii) cognitive function, (viii) smoking, (ix) social engagement, and (x) social support.^16^ The mental health status section consists of only one question on life satisfaction: ‘Are you basically satisfied with your life?’ The response options include ‘Satisfied’, ‘Somewhat satisfied’, ‘Somewhat unsatisfied’, and ‘Unsatisfied’.

### Study design and participant selection

This retrospective cohort study considered data between April 2014 and August 2021. The index date for each individual was defined as the date of their first health checkup during the observation period. Inclusion criteria were individuals who: (i) underwent health checkups for adults aged ≥75 years; (ii) provided complete responses to the mental health status section of the Late‐Stage Elderly Questionnaire; and (iii) were enrolled in the DeSC database at least 1 year prior to the health checkup, enabling a retrospective 1‐year observation period before the index date. We excluded those who had been diagnosed with major depression (ICD‐10 codes: F32 and F33)^21^, ^22^ within 1 year before the index date. Eligible individuals were tracked from their index dates until the occurrence of major depression, disenrollment from the DeSC database, death, end of the study period, or 1 year following the index date, whichever came first.

### Models to detect depression

We developed three models to investigate the sensitivity and specificity of each response in detecting future major depression. In Model 1, individuals who responded ‘Satisfied’ to the question were considered not to have suspected depression, while all others were considered to have suspected depression. In Model 2, those who responded ‘Satisfied’ or ‘Somewhat satisfied’ were considered not to have suspected depression, while others were considered to have suspected depression. In Model 3, those who responded ‘Satisfied’, ‘Somewhat satisfied’, or ‘Somewhat unsatisfied’ were considered not to have suspected depression, while those who responded ‘Unsatisfied’ were considered to have suspected depression. Model 2 incorporates the cut‐off value recommended by the utilization guide of the index test.^16^

### Primary outcome

The primary outcome in this study was major depression within 1 year of the health checkup, defined as diagnosis (ICD‐10 codes: F32 and F33) with the use of antidepressants (ATC code: N06A).^21^, ^22^

### Other variables

We collected data on age, sex, body mass index, smoking status (current or former smoker), the Charlson Comorbidity Index,^23^ comorbidities related to major depression that occurred within 365 days prior to the index date (including organic mental disorders (ICD‐10 code F0), substance use disorders (F1), schizophrenia (F2), stress‐related disorders (F4), eating and sleep disorders (F5x), and other psychiatric disorders), medications related to major depression, and involuntary hospitalization.^21^, ^22^, ^24^ The following medications related to major depression were identified within 30 days before the index date^25^: antidepressants, antipsychotics (multi‐acting receptor targeted antipsychotics, dopamine partial agonist, serotonin dopamine antagonist, phenothiazine, butyrophenone, benzamide, and other antipsychotics), antiepileptics (valproate, lamotrigine, and others), lithium, and hypnotics (benzodiazepines, ramelteon, suvorexant, and others).

### Statistical analysis

The baseline characteristics were summarized to compare the occurrence of major depression using chi‐square tests for categorical variables and *t*‐tests for continuous variables.

We calculated C‐statistics, expressed as the area under the receiver operating characteristic curve, to assess the ability of the questionnaire to screen for major depression. According to a previous report, C‐statistics are classified into poor, acceptable, excellent, and outstanding performance (in this case, screening ability) with ranges of 0.5 to <0.7, 0.7 to <0.8, 0.8 to <0.9, and 0.9 to <1.0, respectively.^26^

Additionally, we assessed the sensitivity, specificity, positive predictive value (PPV), negative predictive value (NPV), and Kappa coefficient for each model to assess the ability to screen for major depression within 1 year.

The threshold for statistical significance was set at *P* = 0.05. All statistical analyses were performed using Stata SE software (version 17.0; StataCorp, College Station, TX, USA). We adhered to the guidelines outlined in the Standards for Reporting of Diagnostic Accuracy Studies (STARD) 2015 statement.^27^

## RESULTS

We identified 11 809 individuals who met the inclusion criteria for the study. After excluding 692 individuals with a prior diagnosis of major depression, 11 117 older adults were included in the analyses. Major depression was found to have newly occurred in 77 (0.7%) individuals, and 45 (0.4%) individuals died.

Table 1 details the baseline characteristics of older adults with and without major depression. Body mass index was missing in six older adults without depression; no other missing data were observed. Older adults with major depression were more likely to be female, have mental comorbidities, and use antipsychotic medications than were those without major depression. They were also were less likely to respond ‘Satisfied’ to the mental health status question.

**Table 1:** Patient characteristics

	Total *N* = 11 117		Without major depression *N* = 11 040		With major depression *N* = 77		*P*‐value
	*n*	(%)	*n*	(%)	*n*	(%)	
Age, years, mean (standard deviation)	80.9	(4.0)	80.9	(4.0)	81.7	(5.0)	0.10
Female	6254	(54.1)	5978	(53.5%)	57	(69.9%)	<0.001
Body mass index, kg/m^2^, mean (standard deviation)	22.9	(3.2)	22.9	(3.2)	22.4	(3.4)	0.130
Smoking habit							0.035
Current smoker	424	(3.8)	420	(3.8%)	4	(5.2%)	
Past smoker	2139	(19.3)	2133	(19.3%)	6	(7.8%)	
Charlson Comorbidity Index, mean (standard deviation)	1.5	(1.7)	1.5	(1.7)	1.8	(1.8)	0.150
Other comorbidities							
Organic mental disorders	542	(4.9)	533	(4.8%)	9	(11.7%)	0.005
Substance use disorder	22	(0.2)	22	(0.2%)	0	(0.0%)	0.690
Schizophrenia	89	(0.8)	84	(0.8%)	5	(6.5%)	<0.001
Stress‐related disorders	1247	(11.2)	1229	(11.1%)	18	(23.4%)	<0.001
Eating disorders and sleep disorders	47	(0.4)	47	(0.4%)	0	(0.0%)	0.570
Other psychiatric disorders	28	(0.3)	27	(0.2%)	1	(1.3%)	0.066
Medications							
Antipsychotics							
Multi‐acting receptor targeted antipsychotic	18	(0.2)	16	(0.1%)	2	(2.6%)	<0.001
Dopamine partial agonist	5	(0.0)	5	(0.0%)	0	(0.0%)	0.850
Serotonin dopamine antagonist	15	(0.1)	15	(0.1%)	0	(0.0%)	0.750
Phenothiazine	8	(0.1)	8	(0.1%)	0	(0.0%)	0.810
Butyrophenone	3	(0.0)	3	(0.0%)	0	(0.0%)	0.880
Benzamide	27	(0.2)	25	(0.2%)	2	(2.6%)	<0.001
Other antipsychotics	2	(0.0)	2	(0.0%)	0	(0.0%)	0.910
Antiepileptics							
Valproate	18	(0.2)	18	(0.2%)	0	(0.0%)	0.720
Lamotrigine	1	(0.0)	1	(0.0%)	0	(0.0%)	0.930
Other antiepileptics	173	(1.6)	173	(1.6%)	0	(0.0%)	0.270
Lithium	4	(0.0)	4	(0.0%)	0	(0.0%)	0.870
Hypnotics							
Benzodiazepine	953	(8.6)	942	(8.5%)	11	(14.3%)	0.072
Ramelteon	30	(0.3)	29	(0.3%)	1	(1.3%)	0.081
Suvorexant	135	(1.2)	133	(1.2%)	2	(2.6%)	0.270
Other hypnotics	542	(4.9)	536	(4.9%)	6	(7.8%)	0.230
Involuntary hospitalization	0	(0.0)	0	(0.0%)	0	(0.0%)	NA
Mental health status section of the Late‐Stage Elderly Questionnaire							0.010
Satisfied	5078	(45.7)	5053	(45.8%)	25	(32.5%)	
Somewhat satisfied	5078	(45.7)	5040	(45.7%)	38	(49.4%)	
Somewhat unsatisfied	841	(7.6)	829	(7.5%)	12	(15.6%)	
Unsatisfied	120	(1.1)	118	(1.1%)	2	(2.6%)	

Abbreviation: NA, not applicable.

**Figure 1:**
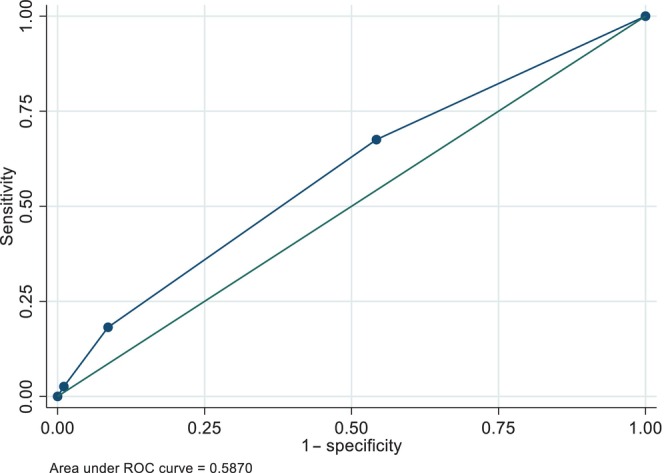
Receiver operating characteristic curve of the ability of a question from the mental health status section of the Late‐Stage Elderly Questionnaire to screen for major depression among participants aged ≥75 years.

Figure 1 depicts the receiver operating characteristic curve evaluating the screening ability of the mental health status section of the Late‐Stage Elderly Questionnaire. The C‐statistic was 0.587.

Table 2 depicts the C‐statistic, sensitivity, specificity, PPV, NPV, and Kappa coefficients for each model. Model 1 demonstrated the highest sensitivity of 0.675 and the lowest specificity of 0.458, whereas Model 3 demonstrated the lowest sensitivity of 0.026 and the highest specificity of 0.989. Model 2 demonstrated a sensitivity of 0.182 and a specificity of 0.914.

**Table 2:** Sensitivity, specificity, positive predictive value, negative predictive value, C‐statistic, and kappa coefficient for each model

	C‐statistic	95% confidence interval	Sensitivity	Specificity	PPV	NPV	Kappa coefficient
Model 1	0.567	0.557–0.576	0.675	0.458	0.009	0.995	0.003
Model 2	0.548	0.539–0.557	0.182	0.914	0.015	0.994	0.014
Model 3	0.508	0.498–0.517	0.026	0.989	0.017	0.993	0.012

Model 1 distinguished between those who responded ‘Satisfied’ and the rest. Model 2 distinguished between those who responded ‘Satisfied’ or ‘Somewhat satisfied’ and the rest. Model 3 distinguished between those who responded ‘Satisfied’, ‘Somewhat satisfied’, or ‘Somewhat unsatisfied’ and ‘Unsatisfied’.

Abbreviations: NPV, negative predictive value; PPV, positive predictive value.

## DISCUSSION

Through this retrospective cohort study, we sought to evaluate the ability of a question in the mental health status section of the Late‐Stage Elderly Questionnaire to screen for major depression in older adults. We observed its poor screening ability, reflected by a C‐statistic of 0.587. Distinguishing the responses of ‘Satisfied’ and ‘Unsatisfied’ from the rest achieved the highest sensitivity (0.675) and highest specificity (0.989), respectively.

Our results indicated three major problems with the question, which was originally intended to screen for major depression. First, the C‐statistics of the questionnaire showed poor screening ability.^26^ Second, the sensitivity for screening major depression may not have been sufficiently high, as Model 1 demonstrated a sensitivity of 0.675, overlooking approximately one‐third of older adults with major depression. Previous studies have advised against the routine use of a single question for screening major depression due to its high rate of overlooking depressed patients.^12^, ^13^ Third, it may result in neglecting untreated major depression. The guide for this questionnaire recommends considering the risk of major depression for responses indicating ‘Somewhat unsatisfied’ or ‘Unsatisfied’.^16^ In the present study, Model 2 showed a sensitivity of 0.182 and a specificity of 0.914. The ‘yea‐saying bias’^28^ may have influenced the results, as older adults who respond ‘Somewhat satisfied’ or ‘Somewhat unsatisfied’ may not consistently provide uniform responses.

These problems may lead to the incorrect classification of older adults as non‐depressed. Thus, questions should be formulated and refined to minimize the risk of neglecting untreated major depression in older adults. For example, it would be beneficial to refer to the Japanese version of the GDS‐15, despite a previous study highlighting the inaccuracies of translation. For example, the word ‘basically’ in the original Item 1 was omitted in translation, leading to errors that resulted in an increased number of negative responses for this item.^29^ Such problems should be solved.

Our study had some limitations. First, health checkups are not mandatory, and those who undergo regular health checkups may be healthier, potentially resulting in selection bias. However, the prevalence of major depression in this study was comparable to that reported in Japan by a previous study (3.0%).^30^ Second, because our study exclusively included Japanese residents, the generalizability of the results to other nations may be limited.

In conclusion, our results suggest that the question in the mental health status section of the Late‐Stage Elderly Questionnaire may not be useful for screening major depression in older adults and requires further refinement.

## AUTHOR CONTRIBUTIONS

SS designed the research; SS, YS, and AO conducted the research; SS, YS, and AO analysed the data; SS, YS, AO, and HY wrote the manuscript; SS was primarily responsible for the final content. All the authors have read and approved the final version of the manuscript.

## ETHICS STATEMENT

This study was approved by the Institutional Review Board of the Graduate School of Medicine at the University of Tokyo. The requirement for written consent was waived owing to data anonymity.

## Data Availability

The datasets analysed in the current study are commercially available, DeSC Healthcare, Inc. providing the database.
